# Processing speed and memory test performance are associated with different brain region volumes in Veterans and others with progressive multiple sclerosis

**DOI:** 10.3389/fneur.2023.1188124

**Published:** 2023-06-08

**Authors:** Rebecca I. Spain, Andrea Hildebrand, Carin S. Waslo, William D. Rooney, Joshua Emmons, Daniel L. Schwartz, Mark S. Freedman, M. Mateo Paz Soldan, Pavle Repovic, Andrew J. Solomon, John Rinker, Mitchell Wallin, Jodie K. Haselkorn, Olaf Stuve, Robert H. Gross, Aaron P. Turner

**Affiliations:** ^1^Department of Veterans Affairs Portland Health Care System, Portland, OR, United States; ^2^Neurology, Oregon Health & Science University, Portland, OR, United States; ^3^Biostatistics and Design Program, Oregon Health & Science University/Portland State University School of Public Health, Portland, OR, United States; ^4^Advanced Imaging Research Center, Oregon Health & Science University, Portland, OR, United States; ^5^Department of Medicine, University of Ottawa and the Ottawa Hospital Research Institute, Ottawa, ON, Canada; ^6^Department of Veterans Affairs, Salt Lake City Health Care System, Salt Lake City, UT, United States; ^7^Neurology, University of Utah, Salt Lake City, UT, United States; ^8^Neurology, Swedish Medical Center, Seattle, WA, United States; ^9^Lerner College of Medicine at the University of Vermont, Burlington, VT, United States; ^10^Neurology, University of Alabama at Birmingham, Birmingham, AL, United States; ^11^Department of Veterans Affairs Washington DC Medical Center, Washington, DC, United States; ^12^University of Maryland School of Medicine, Baltimore, MD, United States; ^13^Department of Veterans Affairs, Puget Sound Health Care System, Seattle, WA, United States; ^14^Rehabilitation Medicine & Epidemiology, University of Washington, Seattle, WA, United States; ^15^Department of Veterans Affairs North Texas Health Care System-Dallas, Dallas, TX, United States; ^16^Neurology, University of Texas Southwestern Medical Center, Dallas, TX, United States; ^17^Peter O'Donnell Brain Institute, University of Texas Southwestern Medical Center, Dallas, TX, United States; ^18^Neurology, University of Colorado Anschutz Medical Campus, Aurora, CO, United States

**Keywords:** progressive multiple sclerosis, veterans, processing speed, verbal memory, visual memory, clinical trials, brain volume changes, brain atrophy

## Abstract

**Background:**

Cognitive dysfunction and brain atrophy are both common in progressive multiple sclerosis (MS) but are seldom examined comprehensively in clinical trials. Antioxidant treatment may affect the neurodegeneration characteristic of progressive MS and slow its symptomatic and radiographic correlates.

**Objectives:**

This study aims to evaluate cross-sectional associations between cognitive battery components of the Brief International Cognitive Assessment for Multiple Sclerosis with whole and segmented brain volumes and to determine if associations differ between secondary progressive (SPMS) and primary progressive (PPMS) MS subtypes.

**Design:**

The study was based on a baseline analysis from a multi-site randomized controlled trial of the antioxidant lipoic acid in veterans and other people with progressive MS (NCT03161028).

**Methods:**

Cognitive batteries were conducted by trained research personnel. MRIs were processed at a central processing site for maximum harmonization. Semi-partial Pearson's adjustments evaluated associations between cognitive tests and MRI volumes. Regression analyses evaluated differences in association patterns between SPMS and PPMS cohorts.

**Results:**

Of the 114 participants, 70% had SPMS. Veterans with MS made up 26% (*n* = 30) of the total sample and 73% had SPMS. Participants had a mean age of 59.2 and sd 8.5 years, and 54% of them were women, had a disease duration of 22.4 (sd 11.3) years, and had a median Expanded Disability Status Scale of 6.0 (with an interquartile range of 4.0–6.0, moderate disability). The Symbol Digit Modalities Test (processing speed) correlated with whole brain volume (*R* = 0.29, *p* = 0.01) and total white matter volume (*R* = 0.33, *p* < 0.01). Both the California Verbal Learning Test (verbal memory) and Brief Visuospatial Memory Test-Revised (visual memory) correlated with mean cortical thickness (*R* = 0.27, *p* = 0.02 and *R* = 0.35, *p* < 0.01, respectively). Correlation patterns were similar in subgroup analyses.

**Conclusion:**

Brain volumes showed differing patterns of correlation across cognitive tasks in progressive MS. Similar results between SPMS and PPMS cohorts suggest combining progressive MS subtypes in studies involving cognition and brain atrophy in these populations. Longitudinal assessment will determine the therapeutic effects of lipoic acid on cognitive tasks, brain atrophy, and their associations.

## 1. Introduction

Accelerated brain volume loss is a frequently used imaging surrogate marker of relapse-independent disease progression in multiple sclerosis (MS) (1). Brain atrophy reflects multiple pathophysiological processes including axonal degeneration, neuronal loss, and loss of glial trophic support. Underlying chronic inflammation, mitochondrial dysfunction, oxidative stress, and loss of blood–brain barrier integrity are implicated in driving central nervous system neurodegeneration that occurs in MS faster than from aging alone (2). Because of strong correlations with clinical disease progression, whole brain atrophy is the primary outcome measure in Phase 2 progressive MS clinical trials (3). However, regional atrophy varies by MS phenotype and by the strength of association with clinical worsening (4). Analyses of total and segmented brain volumes are becoming available to researchers and clinicians by automated processing software packages available as open-source software and marketed commercially (5).

A consistent clinical correlation to brain atrophy in MS is cognitive impairment (6). Cognitive dysfunction, present from early relapsing-remitting MS (RRMS), increases in prevalence and severity with disease duration and in secondary progressive (SPMS) and primary progressive (PPMS) MS subtypes (7). Information processing speed deficits are particularly common (8). Deficits in additional cognitive domains including verbal fluency, verbal episodic memory, visuospatial construction, and executive dysfunction further distinguish progressive from relapsing MS, while visuospatial construction deficits may distinguish SPMS from PPMS (9). Rates of decline among cognitive domains also differ by MS phenotype (10). Because of the frequency of cognitive dysfunction in MS and its relevance to health-related quality of life, cognitive assessment and follow-up are recommended components of routine MS clinical monitoring (11).

Information processing speed tests such as the Paced Auditory Serial Addition Test (PASAT) and Symbol Digit Modalities Test (SDMT) are frequently the only cognitive assessment conducted in clinical practice and clinical trials (12). Batteries of cognitive tests are recommended to assess different cognitive domains. The Brief International Cognitive Assessment for MS (BICAMS) battery includes three short tests assessing information processing speed (SDMT), immediate verbal memory (California Verbal Learning Test, Second Edition; CVLT), and immediate visual memory [memory (Brief Visuospatial Memory Test-Revised; BVMT-R) (13)]. While the BICAMS does not test all cognitive domains, the battery was chosen as a balance between high-yield outcomes and administration efficiency. Studies to date of correlations between the BICAMS tests and brain volumes in progressive MS are limited by small sample sizes, inclusion of relapsing MS or limited to a single progressive MS subtype, or do not contain all three BICAMS tests (14, 15).

The objectives of this study were to determine cross-sectional associations between the components of the comprehensive BICAMS cognitive battery with standard whole and segmented brain volumes in people with progressive MS and to determine if the patterns of associations differ between SPMS and PPMS subtypes.

## 2. Materials and methods

### 2.1. Study design

This is a baseline analysis of an ongoing phase 2, double-blind, multi-center, randomized, placebo-controlled trial (RCT) of 1,200 mg daily oral lipoic acid (LA; Pure Encapsulations, Sudbury, MA) as add-on disease-modifying treatment to slow worsening of gait in progressive MS (NCT03161028). Participants were recruited from five Veterans Affairs Medical Centers as part of the MS Centers of Excellence Network, five United States University sites, and one Canadian study site between August 2018 and January 2022.

### 2.2. Participants

Inclusion criteria to the RCT were ages ≥18 years, prior diagnosis of RRMS or PPMS by 2010 revised McDonald criteria (16), current progressive MS defined as relapse-independent disability progression within the previous 2 years either while not taking disease-modifying therapy (DMT) or despite it, and an EDSS score between 3.0 and 6.5 at screening. Exclusion criteria were other medical or neurological conditions that would confound the assessment of gait, use of scheduled corticosteroid treatments in the year prior to enrollment, corticosteroid treatment for relapse within 60 days of enrollment, use of more than small amounts of LA in the prior 2 years, MRI constraints, pregnant or breastfeeding, significant active concurrent illness, uncontrolled or insulin-dependent diabetes, and lack of English fluency preventing the use of patient-reported outcomes.

Participants included in this baseline analysis were those having complete baseline data for all MRI measures and data from at least one cognitive test. Because of study recruitment from VA medical centers, veteran demographics are reported.

### 2.3. Standard protocol approvals, registrations, and patient consents

The study was approved by the Veterans Affairs Central Institutional Review Board (IRB), the University of Utah Single IRB, the University of Vermont IRB, the Swedish Medical Center IRB, and the Ottawa Research Ethics Board. Written consent was obtained from all the participants prior to performing the study procedures. The trial was registered at Clinicaltrials.gov (NCT03161028).

### 2.4. Cognitive tests

The site study staff were trained on how to administer the BICAMS by a psychologist with experience in neuropsychological assessment in people with MS (AT) at the beginning of the study with an in-person session that was recorded for ongoing study staff training. Cognitive testing was performed on the same day as the MRI acquisition. Cognitive testing was completed using the BICAMS which includes the Symbol Digit Modalities Test (SDMT), the California Verbal Learning Test-Second Edition (CVLT) learning trials, and the Brief Visuospatial Memory Test-Revised (BVMT-R) learning trials (17–19). Raw scores were standardized relative to norms in the general population per the test manual guidelines. While the oral SDMT was the preferred format, some study sites used the written format until the error was noted and corrected. SDMT written was standardized for age and education, while SDMT oral was standardized for age, sex, and education (20). CVLT was standardized for age and sex, and BVMT-R was standardized for age only. Standardization yielded Z-scores for SDMT and T-scores for CVLT and BVMT-R. For Z-scores, the reference mean and standard deviation (sd) are 0 and 1, while for T-scores, the reference mean and sd are 50 and 10. A person with a Z-score of −1 is 1 sd below the reference mean, as is a person with a T-score of 40.

### 2.5. MRI acquisition protocol

3T MRI instruments were used to acquire the following anatomical series of the brain: (1) A magnetization prepared T1-weighted 3D sequence using a sagittally oriented gradient echo readout with a 1-mm isotropic spatial resolution and full brain coverage, as the basis for brain atrophy measures. (2) A fluid-attenuated inversion recovery (FLAIR) 3D T2-weighted series using a sagittally oriented turbo spin echo readout with 1 mm isotropic resolution and full brain coverage. Acquisitions across different 3T platforms were harmonized using the ADNI3 protocol (https://adni.loni.usc.edu/adni-3/) as a guide. Intravenous MR contrast was not administered. (3) The American College of Radiology (ACR) phantom scan was acquired within 7 days of each participant scan for quality control according to ACR protocols.

#### 2.5.1. MRI volumetric analyses

MRI analyses used for study outcomes were analyzed by the Advanced Imaging Research Center at OHSU. T1-weighted and FLAIR Digital Imaging and Communications in Medicine (DICOM) image sets were converted to the Network Interface to File Transfer in the Internet (NIFTI) format, followed by signal intensity bias correction, skull stripped, de-noised, and co-registered. Brain volumetric and cortical thickness measures were extracted from image sets using Freesurfer software tools (v7.1.1) (5). The output using these tools of “total brain parenchymal brain volume” is the summation of normal appearing white matter, white matter hyperintensities, cortical and deep gray matter, and brainstem volumes and is referred to in this article as “whole brain volume.” All tissue class volume masks were visually reviewed for gross errors. Additional brain measurements included total normal appearing white matter volume, total gray matter volume, deep gray matter volume, and cortical thickness. Intracranial vault size was estimated using a custom template generated in Montreal Neuroscience Institute (MNI)152 space followed by non-linear back-registration to native space images. Management of multiple MRI platforms and software was conducted by acquiring high-quality scans meeting quality control standards and by the use of a phantom at every study site.

### 2.6. Statistical analysis

Data analyses were completed using R version 4.2.0 and Stata version 15 (21, 22). Data were inspected to ensure normality and lack of extreme outliers.

Demographic variables (Table 1) and baseline cognitive scores and brain volumes (Table 2) were compared between SPMS and PPMS subtypes. *P*-values were obtained using a *t*-test for continuous variables, Fisher's exact test for dichotomous variables, and Mann–Whitney U-test for ordinal variables. We performed semi-partial correlations to characterize the age- and sex-adjusted correlations between each cognitive measure and each MRI measure. To adjust the MRI output, we regressed each MRI measure on age and sex and extracted the residuals. Sex and age adjustments were not made to cognitive scores as they were already standardized scores. Pearson's correlation coefficients between each set of MRI regression residuals and each cognitive measure were calculated. Correlations were calculated for the full sample, as well as for each subtype (SPMS and PPMS) separately. Although total normal appearing white matter volume, total gray matter volume, deep gray matter volume, and cortical thickness are all components of whole brain volume, there were no statistical accountings performed for the *a priori* correlations between whole brain volume and sub-volumes. Scatterplots visualizing these correlations were produced in R using the ggpubr package (23).

**Table 1:** Participant demographics for the full sample and by secondary progressive and primary progressive multiple sclerosis subtypes.

	**Full sample (*n* = 114)**	**SPMS (*n* = 80, 70%)**	**PPMS (*n* = 34, 30%)**	***p*-value**
Age (years): mean (sd)	59.2 (8.5)	58.6 (8.2)	60.4 (9.2)	0.32
Female: *n* (%)	62 (54.4%)	48 (60.0%)	14 (41.2%)	0.10
White race: *n* (%)^a^	104 (91.2%)	72 (90.0%)	32 (94.1%)	0.72
Ever smoked: *n* (%)	53 (46.5%)	37 (46.3%)	16 (47.1%)	>0.99
Bachelor's degree or higher: *n* (%)	58 (50.9%)	42 (52.5%)	16 (47.1%)	0.81
Duration of disease since first symptom onset (years): mean (sd)	22.4 (11.3)	25.0 (11.0)	16.2 (9.5)	< 0.01
EDSS: median (IQR)	6.0 (4.0–6.0)	6.0 (4.0–6.5)	5.5 (3.6–6.0)	0.06
Clinical and/or radiographic relapse in the 5 years prior to entry: *n* (%)	27 (23.7%)	18 (22.5%)	9 (26.5%)	0.63
Taking DMT: *n* (%)	63 (55.3%)	45 (56.3%)	18 (52.9%)	0.84
Interferons/glatiramer/teriflunomide: *n* (%)^b^	12 (19.0%)	11 (24.4%)	1 (5.6%)	0.15
DMF/fingolimod: *n* (%)^b^	14 (22.2%)	11 (24.4%)	3 (16.7%)	0.74
CD20 B cell therapy: *n* (%)^b^	32 (50.8%)	18 (40.0%)	14 (77.8%)	0.01
Natalizumab: *n* (%)^b^	5 (7.9%)	5 (11.1%)	0 (0.0%)	0.31

a Other races were Black (4.4%), more than one race (2.6%), Asian (0.9%), Unknown (0.9%).

b Percentage of those taking DMT.

DMF, dimethyl fumarate; DMT, disease-modifying therapy; EDSS, Expanded Disability Status Scale; IQR, interquartile range; PPMS, primary progressive multiple sclerosis; SPMS, secondary progressive multiple sclerosis; sd, standard deviation.

**Table 2:** Baseline cognitive scores and MRI brain volumes for the full sample and by secondary progressive and primary progressive multiple sclerosis subtypes.

	**Full sample (*n* = 114)**	**SPMS (*n* = 80, 70.2%)**	**PPMS (*n* = 34, 29.8%)**	***p*-value**
SDMT Z-score (mean, sd)	−1.11 (1.52)	−1.14 (1.56)	−1.05 (1.46)	0.77
CVLT T-score (mean, sd)	53.75 (11.92)	53.96 (10.57)	53.24 (14.81)	0.80
BVMT-R T-score (median, IQR)^a^^,^^b^	44 (34–56)	44 (32.75–56.25)	45 (36–52)	0.78
Proportion with at least 1 impaired cognitive test^c^	48.2% (55)	48.8% (39)	47.0% (16)	>0.99
WBV (mL): mean (sd)	1053.39 (123.58)	1046.51 (128.52)	1069.58 (111.20)	0.34
Total gray matter vol (mL): mean (sd)	465.92 (58.49)	463.39 (62.74)	471.88 (47.30)	0.43
Deep gray matter vol (mL): mean (sd)	47.33 (5.05)	47.03 (5.38)	48.05 (4.15)	0.28
Total white matter vol (mL): mean (sd)	441.29 (73.97)	437.48 (78.70)	450.26 (61.56)	0.36
Mean cortical thickness (mm): mean (sd)	2.34 (0.21)	2.35 (0.22)	2.31 (0.17)	0.40

a BVMT-R sample size was *n* = 113 (80 SPMS, 33 PPMS).

b Mann–Whitney U-test was used for tests of the median due to the assignment of 19 for two participants getting BVMT-R scores at the lower bound of the standardization algorithm, giving them each a T-score of <20 rather than an exact T-score.

c Impaired defined as more than 1.5 sd below the standardized population mean.

BVMT-R, Brief Visuospatial Memory Test-Revised; CVLT, California Verbal Learning Test, second edition; IQR, interquartile range; mL, milliliter; mm, millimeter; PPMS, primary progressive multiple sclerosis; SPMS, secondary progressive multiple sclerosis; sd, standard deviation; SDMT, Symbol Digit Modalities Test; Vol, volume; WBV, whole brain volume.

We also built age- and sex-adjusted linear regression models to characterize the strength of association between each cognitive measure and each MRI measure. Models including the MS subtype and the interaction between the MS subtype and each MRI measure were built to examine the association in each MS subtype independently. Reduced models, not including any MS subtype effects, were built to examine each association in the pooled population (SPMS and PPMS combined).

To adjust for multiple comparisons, we calculated Benjamini–Hochberg adjusted *p*-values with an overall false detection rate of 0.05. Both raw and adjusted *p*-values are presented in tables and plots, but the *p*-values mentioned in the body of the study are adjusted *p*-values.

### 2.7. Data availability

The datasets generated during the current study are not publicly available due to the ongoing status of the longitudinal study but are available from the corresponding author upon reasonable request.

## 3. Results

Of the original study sample size of 115 respondents, one participant was excluded from the analysis because excessive T2 lesion volume resulted in unreliable volume measurements. Participant demographics are listed in Table 1. Mean age was 59.2 (range: 34–73, sd 8.5) years, 54% were women, with mean disease duration of 22.4 (range: 3–49, sd 11.3) years, and median EDSS of 6.0 [interquartile range (IQR) 4.0–6.0]. Twenty-seven (23.7%) participants had active disease defined as clinical or radiographic (new, enlarging, or enhancing MRI lesions) relapses in the 5 years prior to study entry. Most (55.3%) were taking DMT at the baseline visit. The SPMS cohort (*n* = 80, 70.2%) had a longer duration of disease since the first MS symptom onset (mean 25.0 sd 11.0 vs. 16.2 sd 9.5 years, *p* < 0.01). The PPMS cohort had a higher proportion of those on DMT taking B cell-depleting therapies (77.8% vs. 40%, *p* = 0.01). Otherwise, MS subtypes were comparable. Veterans with progressive MS comprised 26% (*n* = 30) of the total sample, of whom 73.3% had SPMS. Mean age of veterans was 58.7 years (range: 34–73, sd 8.9), 23% were women, disease duration was 25.5 years (range: 3–49, sd 12.5), and median EDSS is 5.75 (IQR, 4.0–6.0). Aside from fewer women, veteran demographics and the proportion of SPMS were generally similar to that of the full sample.

Baseline cognitive scores and whole brain and segmented brain volumes including cortical thickness for the full sample and by MS subtype are shown in Table 2. Two participants had BVMT-R scores at the lower bound of the standardization algorithm, giving them T-scores of <20 rather than exact T-scores. Because of this, BVMT-R summary statistics are presented as median and IQR rather than mean and sd. For correlation and regression analyses, these individuals were assigned a T-score of 19. Baseline cognitive scores were similar between SPMS and PPMS subtypes. Defining impairment as scoring more than 1.5 sd below the standardized population mean, 48.2% of all participants (*n* = 55) were impaired on at least one of the three cognitive tests. Half (53.3%) of veterans had an impaired cognitive test. For individual tests, 36.8% (*n* = 42) of all participants had impaired scores for SDMT, 4.4% (*n* = 5) for CVLT, and 25.7% (*n* = 29) for BVMT-R (24).

**Figure 1:**
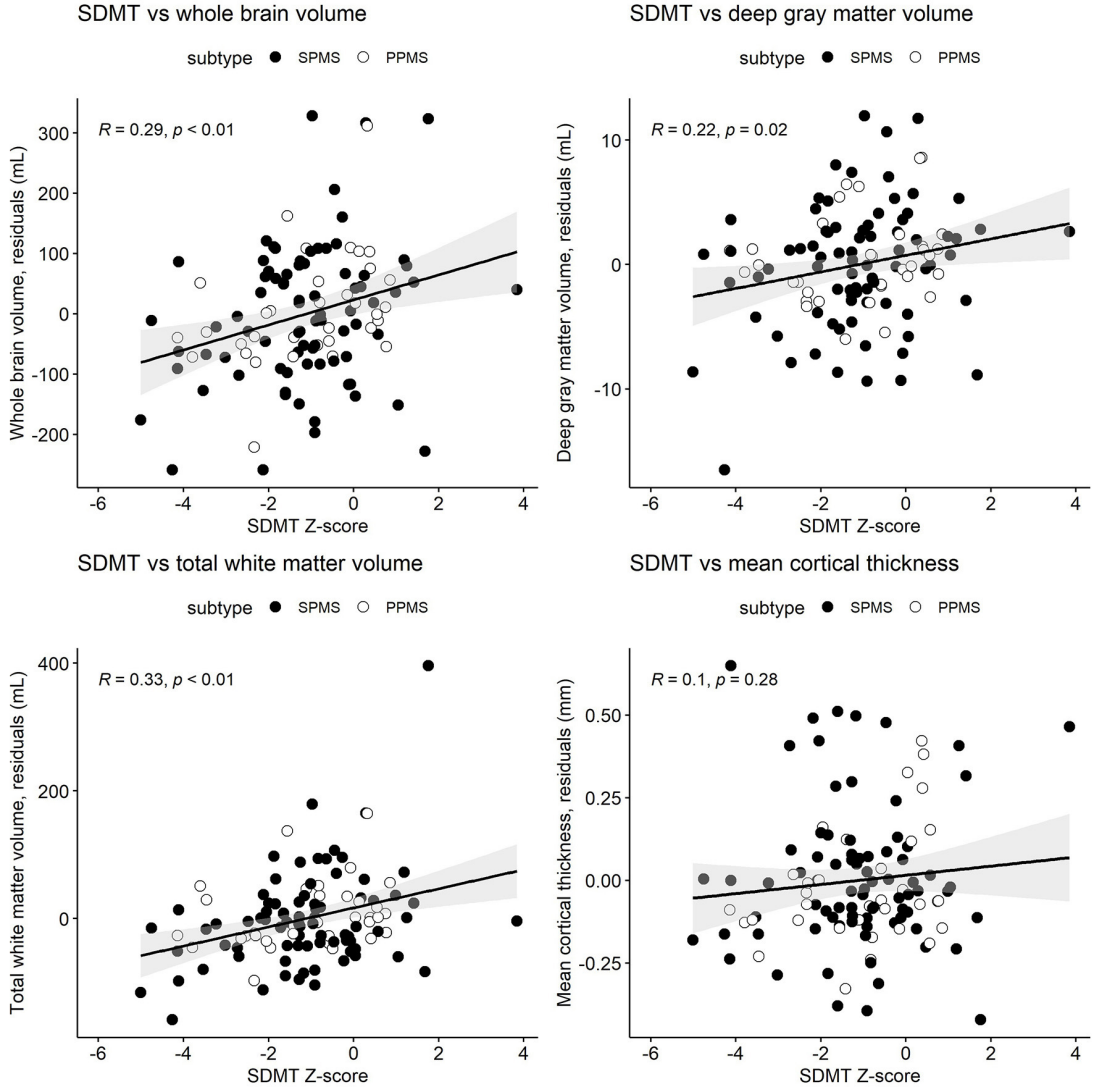
Semi-partial Pearson's correlations between the cognitive processing speed test Symbol Digit Modalities Test (SDMT) Z-scores and whole brain volume, deep gray matter volume, total white matter volume, and mean cortical thickness. MRI measures have been adjusted for age and sex, so what is presented here are the residuals (distance each participant is from the age- and sex-adjusted mean). Correlations are for combined secondary progressive (SPMS, filled circles) and primary progressive (PPMS, unfilled circles) multiple sclerosis cohorts. Statistical significance was set at a *p*-value of ≤ 0.05.

**Figure 2:**
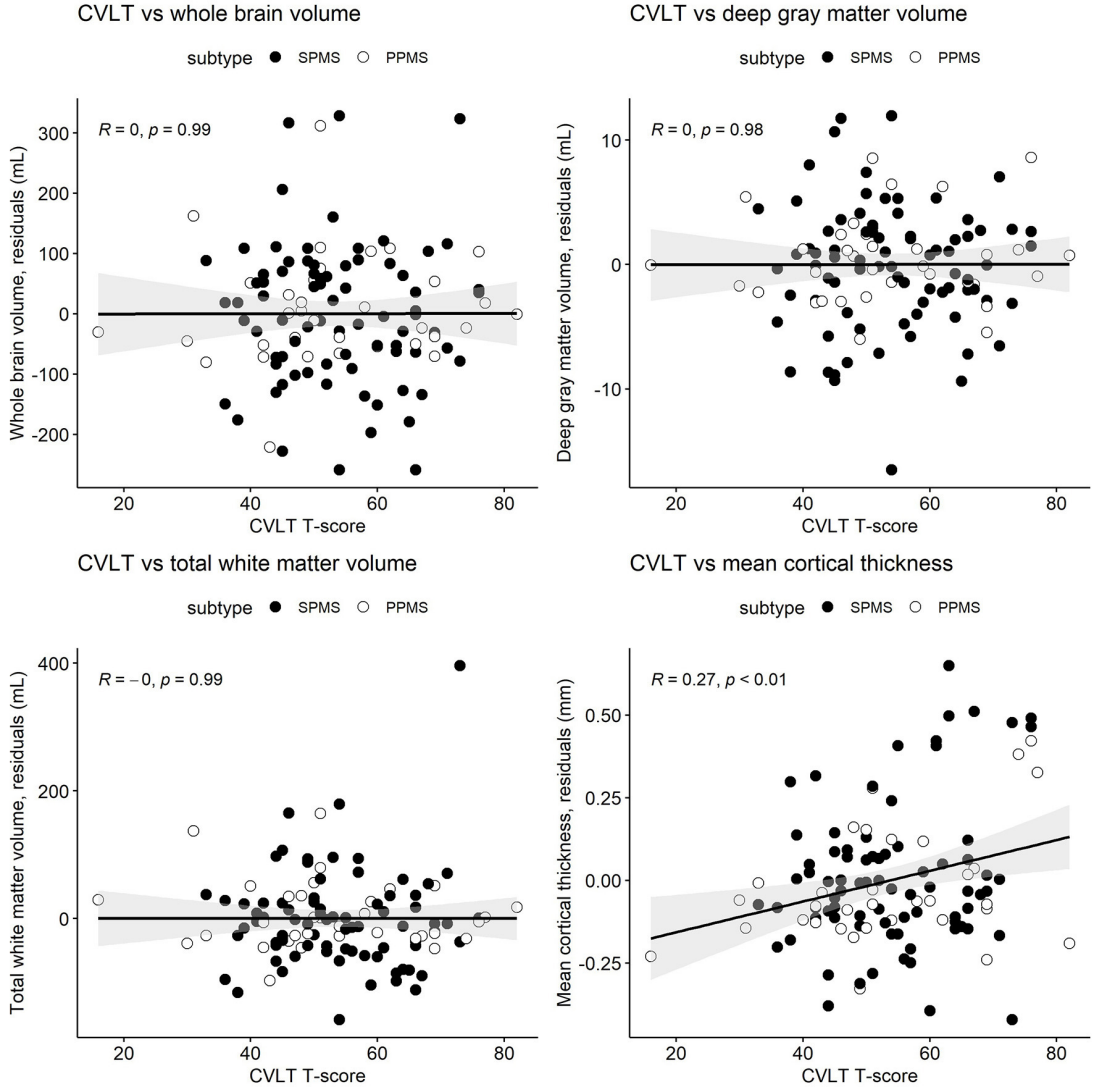
Semi-partial Pearson's correlations between the California Verbal Learning Test, second edition (CVLT) T-scores and whole brain volume, deep gray matter volume, total white matter volume, and mean cortical thickness. MRI measures have been adjusted for age and sex, so what is presented here are the residuals (distance each participant is from the age- and sex-adjusted mean). Correlations are for combined secondary progressive (SPMS, filled circles) and primary progressive (PPMS, unfilled circles) multiple sclerosis cohorts. Statistical significance was set at a *p*-value of ≤ 0.05.

**Figure 3:**
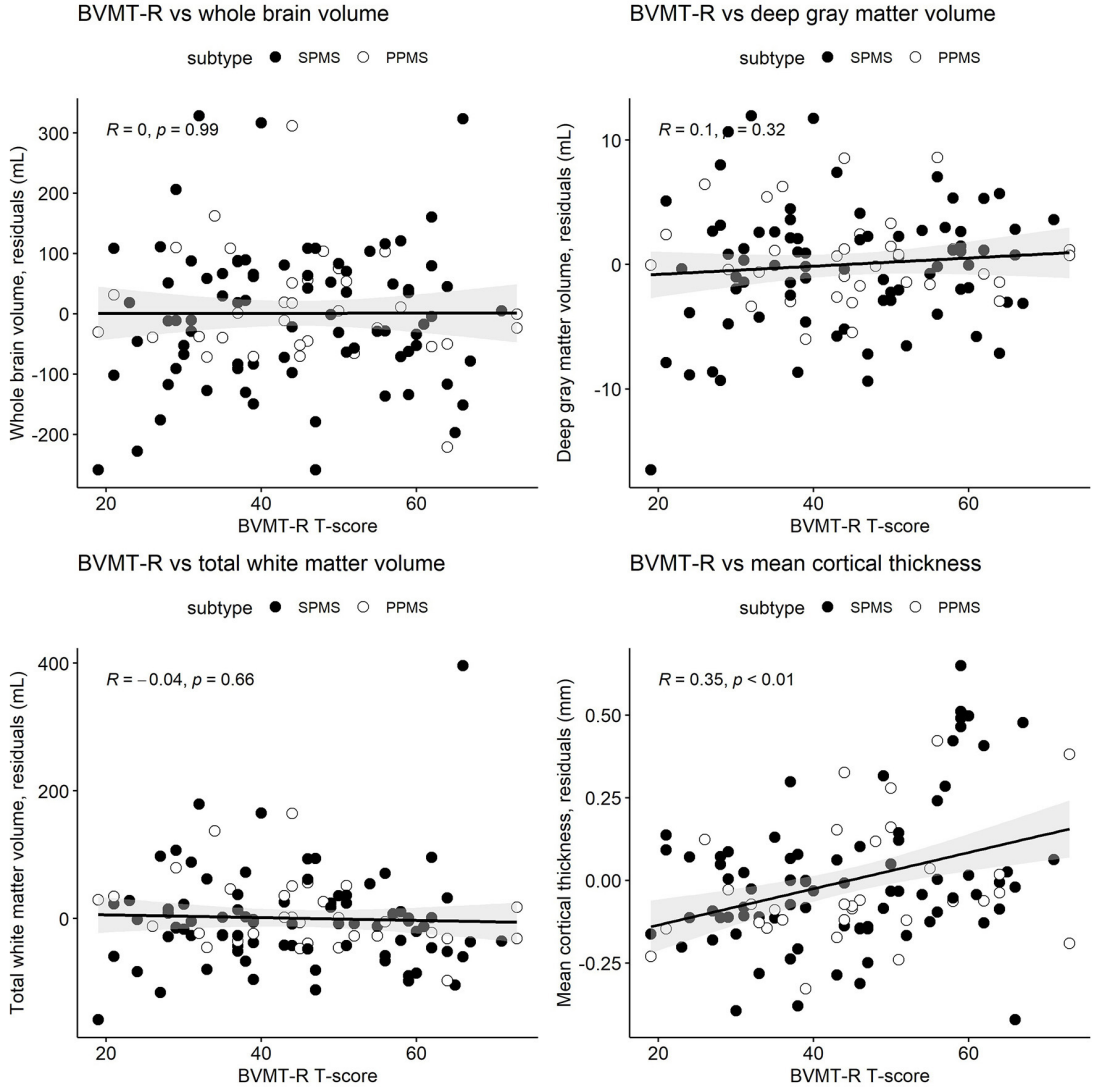
Pearson's correlations between the Brief Visuospatial Memory Test-Revised (BVMT-R) T-scores and whole brain volume, deep gray matter volume, total white matter volume, and mean cortical thickness. MRI measures have been adjusted for age and sex, so what is presented here are the residuals (distance each participant is from the age- and sex-adjusted mean). Correlations are for combined secondary progressive (SPMS, filled circles) and primary progressive (PPMS, unfilled circles) multiple sclerosis cohorts. Statistical significance was set at a *p*-value of ≤ 0.05.

Table 3 and Figures 1–3 present semi-partial Pearson's correlations between cognitive tests and brain volumes with raw and Benjamini–Hochberg adjusted *p*-values. SDMT correlated modestly yet significantly with whole brain volume (*R* = 0.29, *p* = 0.01) and total white matter (*R* = 0.33, *p* < 0.01) (Figure 1, Table 3). SDMT had a smaller correlation with deep gray matter volume, losing significance after adjustment for multiple comparisons (*R* = 0.22, *p* = 0.06). SDMT had smaller and non-significant correlations with total gray matter volume (*R* = 0.13, *p* = 0.46) and mean cortical thickness (*R* = 0.10, *p* = 0.60). In contrast, both the CVLT and BVMT-R correlated modestly and significantly with mean cortical thickness (*R* = 0.27, *p* = 0.02, and R = 0.35, *p* < 0.01, respectively) but not with total brain parenchymal volume, total gray matter volume, or total white matter volume (all *p* ≥ 0.60) (Figures 2, 3, Table 3).

**Table 3:** Semi-partial Pearson's correlations (R) between cognitive tests and sex- and age-adjusted brain volumes for the full sample of participants and by secondary progressive and primary progressive MS subtypes.

**Correlation pairings**	**Full sample (*****n*** = **114)**		**SPMS (*****n*** = **80, 70.2%)**		**PPMS (*****n*** = **34, 29.8%)**	
	***R***	**p**_raw_ **/p**_adj_	**R**	**p**_raw_ **/p**_adj_	**R**	**p**_raw_ **/p**_adj_
**SDMT**						
Whole brain vol	0.29	**< 0.01/** ***0.01***	0.26	**0.02**/0.10	0.39	**0.03**/0.12
Total gray matter vol	0.13	0.18/0.46	0.05	0.69/0.85	0.40	**0.02**/0.12
Deep gray matter vol	0.22	**0.02**/0.06	0.22	**0.04**/0.15	0.20	0.27/0.45
Total white matter Vol	0.33	**< 0.01/** *** < 0.01***	0.34	**< 0.01/** ***0.01***	0.27	0.12/0.22
Mean cortical thickness	0.10	0.28/0.60	0.03	0.79/0.81	0.34	**0.05**/0.19
**CVLT**						
Whole brain vol	0.00	0.99/0.99	−0.03	0.77/0.85	0.08	0.64/0.74
Total gray matter vol	0.04	0.70/0.95	−0.08	0.45/0.85	0.32	0.06/0.19
Deep gray matter vol	0.00	0.98/0.99	−0.02	0.85/0.85	0.07	0.71/0.76
Total white matter vol	0.00	0.99/0.99	0.05	0.67/0.85	−0.12	0.50/0.69
Mean cortical thickness	0.27	**< 0.01/** ***0.02***	0.22	**0.05**/0.15	0.39	**0.02**/0.12
**BVMT-R**						
Whole brain vol	0.00	0.99/0.99	0.06	0.62/0.85	−0.18	0.30/0.45
Total gray matter vol	0.08	0.42/0.70	0.09	0.41/0.85	0.02	0.91/0.91
Deep gray matter vol	0.09	0.32/0.60	0.14	0.20/0.51	−0.08	0.64/0.74
Total white matter vol	−0.04	0.66/0.95	0.03	0.82/0.85	−0.30	0.09/0.19
Mean cortical thickness	0.35	**< 0.01/** *** < 0.01***	0.37	**< 0.01/** ***0.01***	0.31	0.08/0.19

BVMT-R, Brief Visuospatial Memory Test-Revised; CVLT, California Verbal Learning Test, second edition; PPMS, primary progressive multiple sclerosis; SPMS, secondary progressive multiple sclerosis; SDMT, Symbol Digit Modalities Test; TBP, Total brain parenchymal; Vol, volume.

Raw (*p*_raw_) and Benjamini–Hochberg adjusted (*p*_adj_) *p*-values presented. Statistical significance was set at a *p*-value of ≤ 0.05.

Regression analyses did not reveal systematic differences between SPMS and PPMS subtypes in the correlations between cognitive tests and brain volume. Associations between cognitive scores and brain volumes are broadly similar within each MS subtype (Supplementary Table 1), and there was no evidence of a consistent subtype interaction effect (Supplementary Table 2).

## 4. Discussion

This study demonstrated that components of the comprehensive BICAMS cognitive battery had unique correlation patterns with brain volumes in people with progressive MS. Specifically, the information processing speed (SDMT) test correlated with normalized whole brain and total white matter volumes, while the verbal memory (CVLT) and visual memory (BVMT-R) tests correlated only with mean cortical thickness. Correlations were low to moderate ranging from *R* = 0.22 to *R* = 0.35. However, the unique patterns of correlations are suggestive of unique CNS pathways driving cognitive domains. While statistical significance suffered due to lower sample sizes, the overall patterns of correlations were similar between SPMS and PPMS cohorts as supported by regression analysis. The similar patterns of correlations support the broad overlap in the pathophysiology of SPMS and PPMS including injury to normal appearing white and gray matter and cortical lesions (25). This suggests combining SPMS and PPMS subtypes when analyzing associations between brain volumes and cognitive tests in the domains assessed. Given similar demographics and proportion with baseline cognitive impairment of veterans to the larger cohort, the conclusions of our analyses may apply to veteran populations.

The strength of correlation between SDMT and whole brain volume (*R* = 0.29) found in our population is slightly less than in similar studies in relapsing and mixed relapsing and progressive MS cohorts. In purely relapsing MS cohorts, Fenu et al. report nearly identical Pearson's correlations between SDMT and whole brain volume (*R* = 0.38, *n* = 195) as D'hooghe et al. (*R* = 0.4, *n* = 254) and Calabrese et al. (*R* = 0.41, *n* = 70) despite different MRI segmentation methodologies (26–28). Interestingly and despite a smaller sample size, Fenu also found significant correlations between SDMT and total gray matter and mean cortical thickness (*R* = 0.31 and *R* = 0.35, respectively), while D'hooghe et al. did not find correlations with additional brain regions. Fenu et al. additionally reported significant correlations between both CVLT and BVMT-R and whole brain, total gray matter, and cortical thickness as reported with SDMT, and with similar correlation strengths (from *R* = 0.24 to *R* = 0.36) (25). In a mixed relapsing and progressive MS cohort, Benedict et al. also reported a modest correlation between SDMT and whole brain volume (*R* = 0.40) but no significant correlations between whole and segmented brain volumes with CVLT or BVMT-R (29).

We found only one study conducted in a purely progressive MS population relating BICAMS to brain volumes. Gueveia et al. reported significant correlations of both the SDMT and BVMT-R with deep gray matter volume (*R* = 0.66 and *R* = 0.41, respectively) in a PPMS cohort (*n* = 55), a finding we did not replicate, along with a significant correlation between BVMT-R and neocortical gray volume (*R* = 0.39) (15). This group did not evaluate whole brain volumes. Comparison of our findings to other progressive MS cohorts is anticipated given the growing numbers of treatment trials for progressive MS.

Differences in methodologies of studies associating cognitive tests and brain volumes in non-MS cohorts including small vessel disease, neuromyelitis optica, traumatic brain injury, and normal aging limit direct comparisons to our results; however, heterogeneous findings suggest a lack of consensus regarding the clinical implications of whole or regional atrophy (30, 31). Brain atrophy in MS may represent a late-stage neurodegenerative phenomenon from demyelination and axonal and glial cell loss. Alternative imaging techniques such as diffusion tensor imaging and MR spectroscopy may detect structural and functional damage prior to irreversible atrophy, highlighting a treatment opportunity (32, 33).

Our participant population, although not selected for cognitive dysfunction, demonstrated a high prevalence (48.2%) of scores more than 1.5 sd below the standardized population mean on cognitive tests, highlighting the importance of identifying and treating cognitive dysfunction in progressive MS. While the SDMT had the highest percentage of abnormal scores (36.8%), 13 participants (11.4%) had abnormal scores in other cognitive domains that would have been missed if the SDMT was the only cognitive test utilized. This reinforces the value of screening across multiple cognitive domains when possible for clinical and research inquiries.

An unexpected finding of our study was the relative lack of impairment in CVLT performance compared to the SDMT and BVMT-R. Usually, impairment is found in all three domains tested in the BICAMS. While neither the CVLT nor BVMT-R gets corrected for education level, our highly educated study population may have had better auditory learning and biased the results. Interestingly, one study found the combination of SDMT and BVMT-R to be the most sensitive to cognitive impairment and had the strongest association with the full battery, suggesting that the CVLT performance may be less clinically relevant (34).

Strengths of the study include the relatively large sample of SPMS and PPMS participants from a large geographical area, trained personnel conducting cognitive testing, centralized blinded scoring of the CVLT and BVMT-R by two raters, and a centralized MRI processing site for brain volume analyses. Study limitations arise primarily from drawing the study sample from an interventional study and not based on the current analyses. The interventional trial required active disability worsening for study entry. As such, our results may not be applicable to stable, non-worsening MS populations. The sample size was not powered to detect differences between SPMS and PPMS in the measures investigated here. The SDMT was administered variously in oral and written formats which, although scored appropriately for the format, may have affected the analyses. The BICAMS battery does not include cognitive domains that may distinguish SPMS and PPMS (9). This analysis lacked healthy control or RRMS comparison populations thereby limiting conclusions about the uniqueness of our findings to progressive MS. Finally, regional cortical thickness and deep gray matter volume—both linked to cognitive dysfunction in some MS studies—were not outcomes of the interventional trial study but are ones that could be investigated in future (4, 35). The cross-sectional design of the current analysis limits conclusions about causality or influence. In fact, longitudinal data from a large interventional trial of natalizumab in SPMS did not find MRI volume changes associated with the worsening of SDMT or other measures of disability (36). Anticipated longitudinal studies including this one in progressive MS populations will clarify the clinical correlates of whole and regional brain atrophy.

## 5. Conclusion

In this cross-sectional study of veterans and other people with progressive MS, information processing speed was associated with whole brain and total white matter volumes, while verbal and visual memory tests were associated with mean cortical thickness. The strengths of associations were all modest though statistically significant. SPMS and PPMS subtypes appeared to have similar patterns of associations although small numbers precluded definitive confirmation. The planned longitudinal examination of this cohort will determine if changes in cognition and brain volume over time are associated. Advanced imaging techniques may determine if other measures are better predictors of cognitive or other disabilities change over time in progressive MS.

## Data availability statement

The datasets presented in this article are not readily available because the datasets generated during and/or analyzed during the current study are not publicly available due to the ongoing status of the longitudinal study, but are available from the corresponding author on reasonable request. The study protocol and statistical analysis plan for the randomized controlled trial has not been attached as a Supplementary File because the methods and analyses for the current cross-sectional analysis are not included in those documents. The protocol and statistical analysis plan for the randomized trial will be included in the primary outcome paper. Requests to access the datasets should be directed to spainr@ohsu.edu.

## Ethics statement

The studies involving human participants were reviewed and approved by VA Central IRB, University of Utah Single IRB, University of Vermont IRB, Swedish Medical IRB, Ottawa Research Ethics Board. The patients/participants provided their written informed consent to participate in this study.

## Author contributions

RS: conceptualization, methodology, investigation, writing, and reviewing and editing. AH: methodology, software, formal analysis, visualization, and writing. CW: investigation and data curation and writing. WR: methodology, data processing, and reviewing and editing. JE and DS: investigation and data processing. MF, MP, PR, AS, JR, MW, JH, OS, and RG: investigation and reviewing and editing. AT: conceptualization, methodology, and reviewing and editing. All authors contributed to the article and approved the submitted version.
