# Supplementary material for: Processing speed and memory test performance are associated with different brain region volumes in Veterans and others with progressive multiple sclerosis
Source: Front Neurol. 2023 Jun 8;14:1188124. doi: 10.3389/fneur.2023.1188124 (PMC10285490; doi:10.3389/fneur.2023.1188124)
Supplement: Supplementary file 1 [file Table_1.pdf]

Appendix Table 1. Regression analyses of the associations between cognitive scores and brain volumes in the full sample and within each MS subtype.

	Full Sample (n=114)		SPMS (n=80)		PPMS (n=34)	
	β (95% CI)	p_{raw} / p_{adj}	β (95% CI)	p_{raw} / p_{adj}	β (95% CI)	p_{raw} / p_{adj}
**SDMT**						
Whole brain vol	0.004 (0.002–0.006)	**<0.01** / ***0.01***	0.004 (0.001 – 0.006)	**0.01** / ***0.05***	0.005 (0.0002 – 0.010)	**0.04** / 0.18
Total gray matter vol	0.004 (-0.002 – 0.009)	0.19 / 0.46	0.002 (-0.004 – 0.008)	0.48 / 0.81	0.011 (-0.001 – 0.023)	0.06 / 0.18
Deep gray matter vol	0.072 (0.012 – 0.132)	**0.02** / 0.06	0.071 (0.005 – 0.137)	**0.03** / 0.13	0.073 (-0.058 – 0.205)	0.27 / 0.37
Total white matter Vol	0.007 (0.003 – 0.011)	**<0.01** / ***<0.01***	0.007 (0.003 – 0.011)	**<0.01** / ***0.01***	0.007 (-0.002 – 0.015)	0.12 / 0.22
Mean cortical thickness	0.74 (-0.62 – 2.10)	0.29 / 0.59	0.26 (-1.27 – 1.79)	0.74 / 0.84	2.66 (-0.36 – 5.68)	0.08 / 0.18
**CVLT**						
Whole brain vol	0.0002 (-0.020 – 0.020)	0.99 / 0.99	-0.006 (-0.028 – 0.016)	0.59 / 0.81	0.023 (-0.016 – 0.062)	0.25 / 0.37
Total gray matter vol	0.009 (-0.035 – 0.052)	0.70 / 0.95	-0.016 (-0.061 – 0.029)	0.49 / 0.81	0.123 (0.035 – 0.211)	**0.01** / ***0.05***
Deep gray matter vol	0.005 (-0.472 – 0.482)	0.98 / 0.99	-0.010 (-0.618 – 0.419)	0.70 / 0.85	0.491 (-0.551 – 1.532)	0.35 / 0.44
Total white matter Vol	-0.0002 (-0.032 – 0.031)	0.99 / 0.99	0.001 (-0.034 – 0.036)	0.95 / 0.95	-0.006 (-0.077 – 0.064)	0.86 / 0.86
Mean cortical thickness	15.14 (4.93 – 25.35)	**<0.01** / ***0.02***	10.30 (-1.09 – 21.69)	0.08 / 0.23	34.47 (12.00 – 56.95)	**<0.01** / ***0.05***
**BVMT-R**						

Whole brain vol	0.0002 (-0.023 – 0.023)	0.98 / 0.99	0.007 (-0.018 – 0.033)	0.56 / 0.81	-0.028 (-0.073 – 0.017)	0.22 / 0.36
Total gray matter vol	0.021 (-0.029 – 0.070)	0.41 / 0.68	0.028 (-0.025 – 0.081)	0.30 / 0.65	-0.018 (-0.124 – 0.088)	0.73 / 0.79
Deep gray matter vol	0.275 (-0.266 – 0.816)	0.32 / 0.59	0.419 (-0.166 – 1.004)	0.16 / 0.40	-0.467 (-1.645 – 0.711)	0.43 / 0.50
Total white matter Vol	-0.008 (-0.044 – 0.028)	0.66 / 0.95	0.005 (-0.034 – 0.044)	0.79 / 0.84	-0.072 (-0.152 – 0.007)	0.07 / 0.18
Mean cortical thickness	23.53 (11.24 – 33.81)	**<0.01** / ***<0.01***	22.92 (10.17 – 35.67)	**<0.01** / ***0.01***	22.83 (-2.33 – 48.00)	0.08 / 0.18

BVMT-R, Brief Visuospatial Memory Test- Revised; CVLT, California Verbal Learning Test, 2nd edition; PPMS, primary progressive multiple sclerosis; SPMS, secondary progressive multiple sclerosis; SDMT, Symbol Digit Modalities Test; TBP, Total brain parenchymal; Vol, volume
