# Supplementary material for: Processing speed and memory test performance are associated with different brain region volumes in Veterans and others with progressive multiple sclerosis
Source: Front Neurol. 2023 Jun 8;14:1188124. doi: 10.3389/fneur.2023.1188124 (PMC10285490; doi:10.3389/fneur.2023.1188124)
Supplement: Supplementary file 2 [file Table_2.pdf]

Appendix Table 2. Regression output for the main effects of each cognitive/MRI association, and the MS subtype interaction effect of each association. MS subtype interaction effect is interpreted as the difference in effect for people with secondary progressive versus primary progressive as the reference level.

	Main effects β (95% CI)	p_{raw} / p_{adj}	Subtype interaction β (95% CI)	p_{raw} / p_{adj}
**SDMT**				
Whole brain vol	0.004 (0.002– 0.006)	**<0.01** / ***0.01***	0.001 (-0.004 – 0.007)	0.61 / 0.83
Total gray matter vol	0.004 (-0.002 – 0.009)	0.19 / 0.46	0.009 (-0.003 – 0.021)	0.16 / 0.34
Deep gray matter vol	0.072 (0.012 – 0.132)	**0.02** / 0.06	0.002 (-0.140 – 0.144)	0.98 / >0.99
Total white matter Vol	0.007 (0.003 – 0.011)	**<0.01** / ***<0.01***	-0.0002 (-0.010 – 0.009)	0.96 / >0.99
Mean cortical thickness	0.74 (-0.62 – 2.10)	0.29 / 0.59	2.40 (-0.98 – 5.80)	0.15 / 0.34
**CVLT**				
Whole brain vol	0.0002 (-0.020 – 0.020)	0.99 / 0.99	0.029 (-0.014 – 0.072)	0.18 / 0.34
Total gray matter vol	0.009 (-0.035 – 0.052)	0.70 / 0.95	0.139 (0.044 – 0.233)	**<0.01** / 0.06
Deep gray matter vol	0.005 (-0.472 – 0.482)	0.98 / 0.99	0.590 (-0.531 – 1.711)	0.30 / 0.50
Total white matter Vol	-0.0002 (-0.032 – 0.031)	0.99 / 0.99	-0.007 (-0.084 – 0.069)	0.85 / >0.99
Mean cortical thickness	15.14 (4.93 – 25.35)	**<0.01** / ***0.02***	24.18 (-1.02 – 49.38)	0.06 / 0.34
**BVMT-R**				
Whole brain vol	0.0002 (-0.023 – 0.023)	0.98 / 0.99	-0.036 (-0.084 – 0.013)	0.15 / 0.34
Total gray matter vol	0.021 (-0.029 – 0.070)	0.41 / 0.68	-0.046 (-0.158 – 0.066)	0.42 / 0.63
Deep gray matter vol	0.275 (-0.266 – 0.816)	0.32 / 0.59	0.886 (-2.152 – 0.380)	0.17 / 0.34
Total white matter Vol	-0.008 (-0.044 – 0.028)	0.66 / 0.95	-0.078 (-0.164 – 0.008)	0.08 / 0.34
Mean cortical thickness	23.53 (11.24 – 33.81)	**<0.01** / ***<0.01***	-0.09 (-28.30 – 28.13)	>0.99 / >0.99

BVMT-R, Brief Visuospatial Memory Test- Revised; CVLT, California Verbal Learning Test, 2nd edition; PPMS, primary progressive multiple sclerosis; SPMS, secondary progressive multiple sclerosis; SDMT, Symbol Digit Modalities, Test; TBP, Total brain parenchymal; Vol, volume
